# Health related quality of life after extremely preterm birth: a matched controlled cohort study

**DOI:** 10.1186/1477-7525-8-53

**Published:** 2010-05-23

**Authors:** Bente J Vederhus, Trond Markestad, Geir E Eide, Marit Graue, Thomas Halvorsen

**Affiliations:** 1 Department of Paediatrics, Haukeland University Hospital, Bergen, Norway; 2 Department of Public Health and Primary Health Care, University of Bergen, Norway; 3 Department of Clinical Medicine, University of Bergen, Norway; 4 Centre for Clinical Research, Haukeland University Hospital, Bergen, Norway; 5 Department of Nursing, Bergen University College, Bergen, Norway

## Abstract

**Background:**

The majority of infants born before the last trimester now grow up. However, knowledge on subsequent health related quality of life (HRQoL) is scarce. We therefore aimed to compare HRQoL in children born extremely preterm with control children born at term. Furthermore, we assessed HRQoL in relation to perinatal and neonatal morbidity and to current clinical and sociodemographic characteristics.

**Method:**

*The Child Health Questionnaire *(CHQ-PF50) and a general questionnaire were applied in a population based cohort of 10 year old children born at gestational age ≤ 28 weeks or with birth weight ≤ 1000 grams in Western Norway in 1991-92 and in term-born controls, individually matched for gender and time of birth. The McNemar test and paired t-tests were used to explore group differences between preterms and matched controls. Paired regression models and analyses of interaction (SPSS mixed linear model) were used to explore potential effects of sociodemographic and clinical characteristics on HRQoL in the two groups.

**Results:**

All 35 eligible preterm children participated. None had major impairments. Learning and/or attention problems were present in 71% of preterms and 20% of controls (odds ratio (OR): 7.0; 95% confidence interval (CI): 2.2 to 27.6). Insufficient professional support was described by 36% of preterm vs. 3% of control parents (OR: infinite; CI: 2.7 to infinite). Preterms scored lower on eight CHQ-PF50 sub-scales and the two summary scores, boys accounting for most of the deficits in areas of behavior, psychosocial functioning and parental burden. HRQoL was associated with learning and/or attention problems in both preterm and control children, significantly more so in preterms in areas related to health and parental burden. Within the preterm group, HRQoL was mostly unrelated to perinatal and neonatal morbidity.

**Conclusions:**

HRQoL for children born extremely preterm, and particularly for boys, was described by parents to be inferior to that of children born at term, and sufficiently poor to affect the daily life of the children and their families. Learning and/or attention problems were reported for a majority of preterms, strongly influencing their HRQoL.

## Background

To the benefit of all seriously ill newborns, substantial improvements have occurred in neonatal intensive care during the last decades. In parallel with this development, the survival rates for extremely preterm infants have increased substantially. If resuscitated, approximately 80% of these infants will grow up [1]. One may envision two possible cohort effects from this scenario: Less sequelae due to better treatment or more sequelae due to increased survival of more vulnerable individuals. Repeated and comprehensive long-term follow-up studies are therefore needed to identify areas of concern in this very special population. In this context, one must bear in mind that preterm birth is not a disease entity in itself, but a risk factor for subsequent functional deficits of partly unknown qualities and quantities. Importantly, few of the disorders that have been linked to preterm birth are specific for this population but may somehow be observed also in children born at term, although less prevalent and often with somewhat different appearances. These issues are not well understood, impeding evidence based adjustments of the neonatal treatment and adequate follow-up measures throughout childhood.

Major disabilities (e.g. cerebral palsy) are generally recognized early, and referred to relevant remedial programs. However, to recognize and foresee the significance of milder impairments may be more difficult [2]. When tested, preterm children generally score poorer on areas related to behavior, emotional health and learning capacity, influencing psychosocial functioning [3-5]. Also with respect to general health, preterm children are at risk of deficits with potential functional consequences, e.g reduced lung function and exercise capacity [6-8]. However, the impact from such deficits on the overall well-being of the child and the family is not well described. A systematic review of health related quality of life (HRQoL) research in children born preterm, concludes that preschool children who are born preterm tend to be scored lower by their parents, but that the literature in school-aged children is scarce and the issue therefore important to address [9]. Interestingly, in the few studies available on adolescents, subjects born preterm report their HRQoL quite similar to peers born at term, while their parents' assessment is significantly lower [10-12].

HRQoL is a multidimensional construct of physical, psychological and functional well-being, i.e. subjective information beyond morbidity, as observed from the perspective of a parent or from the child itself - or better, both [13,14]. Even though quality of life (QoL) and HRQoL are related and often used interchangeably they are not identical, as QoL is a broader concept referring more to a child's feelings and appraisal with his or her life while HRQoL somewhat refers to a child's functional status [15]. Functional status may be defined as "the child's ability to perform daily activities that are essential to meet his or her basic needs, fulfill roles, and maintain health and well-being" (Drotar p. 358) [16].

The aim of the present study was to assess HRQoL in 10 year old children born extremely preterm compared to children born at term, and to assess if and how perinatal and neonatal morbidity and current clinical and sociodemographic characteristics were related to HRQoL.

## Methods

### Subjects

The subjects participated in a comprehensive follow-up study assessing different long-term outcome measures, of which some have been described in detail elsewhere [6]. Briefly, eligible children were born at a gestational age (GA) ≤ 28 weeks or with a birth weight ≤ 1000 g in 1991-1992 within a defined region in Western Norway. Of 47 eligible infants admitted to the NICU, 12 (26%) died (seven girls, three boys, two unknown sex). All the 35 survivors participated in this study. Medical care had been provided at the only regional neonatal intensive care unit (NICU) at Haukeland University Hospital, Bergen.

For each preterm, the temporally nearest term born infant of the same gender with birth weight between 3 and 4 kg (Norwegian 10^th ^to 90^th ^centile) [17] was recruited as control. If one potential control subject declined to participate, the next born subject was approached, and so on until one term born child was recruited for each enrolled preterm.

### Methods

One pediatrician (TH) assessed current health status in all subjects through a standard medical history and physical examination. Current pulmonary function was described by forced expiratory volume in the first second (FEV_1_), measured with Sensor Medics Vmax 22 spirometer (*Anaheim, CA, USA*) and transformed to percentages of predicted with a standard reference equation [18]. All relevant medical information for preterm and control subjects alike, was available from hospital records. Perinatal and neonatal characteristics were described in terms of maternal infection, use of antenatal corticosteroids, birth weight ratio (ratio between birth weight and the 50^th. ^percentile for gestational age) and selected markers of early morbidity, i.e. cerebral hemorrhage, days on mechanical ventilation, and severity of lung disease (severity of bronchopulmonary dysplasia (BPD) and duration of oxygen treatment).

HRQoL was assessed with the *Child Health Questionnaire-Parent Form 50 (CHQ*), a validated, generic instrument that measures functional health and well-being of a child through the eyes of a parent. The physical, emotional and social well-being of the child and the perceived burden of the child's health on the family is addressed [19]. The questionnaire follows the definition of health, given by The World Health Organization as "*a state of complete physical, mental, and social wellbeing and not merely the absence of disease or infirmity*" [20]. Health is assessed over several domains including: general health perceptions, physical functioning, role/social physical functioning, bodily pain, role/social emotional and behavioral functioning, parent impact-time and parent impact-emotional, self-esteem, mental health, behavior, family activities and family cohesion. All scales except family cohesion and general health use a recall period of the preceding four weeks. The responses are indicated along an ordered 4 to 6 point Likert-type scale specifying level of agreement to a certain categorical statement such as "very often" to "not at all". The items within each scale are summarized and linearly transformed into a scale of 0 (poor) to 100 (optimal) for each dimension. The instrument also consists of two summary scores, physical and psychosocial, constructed from factor analyses of ten different sub-scales, and standardized based on means and standard deviation (SD) from a combined US population and linearly transformed, yielding a mean score of 50 and a SD of 10. The Norwegian version of this instrument had been validated in a pediatric population with juvenile arthritis with good internal consistency (Cronbach's alpha = 0.84), and capacity to discriminate towards healthy subjects and to be sensitive to clinical changes [21-23]. Differences of 5-10 points on a 100-point scale are regarded as clinically significant [24].

Information on sociodemographic characteristics and the children's functioning were obtained through the CHQ and a questionnaire to the parents, which was developed for the study. Data included parents' education, parents' assessment of their child's school performance and learning or attention problems as reported to them by health professionals or teachers, participation in sport and social activities, extent of professional, academic and psychological support, perceived adequacy of support and counseling from professional bodies or remedial programs during childhood, and financial support through the National Insurance Scheme. The questionnaires were completed by the parent while the child was examined by health personnel, allowing a relaxed atmosphere and ample time. The same nurse supervised the procedure, assuring that similar information was given to all parents completing the questionnaires.

The study was approved by the Regional Committee on Medical Research Ethics of the Western Norway Health Region and the Norwegian Data Inspectorate. Before enrolment, the National Registry was consulted to ensure that subjects were still living. Informed written consent was obtained from the parents.

### Statistical analyses

Cronbach's alpha was used to determine internal consistency of the CHQ-PF50 scores.

The McNemar test and the t-test for paired samples were used to explore group differences between preterms and matched controls on categorical and continuous demographic variables, respectively. Differences between preterm and control subjects on the CHQ-PF50 scores were examined with the Wilcoxon signed rank test and the paired sample t-test, as appropriate. The mixed linear model [25] was used to study potential differences in HRQoL between the premature and the matched term born control children, adjusted for potential confounders, i.e. gender, physical activity, learning and/or attention problems and FEV_1_. Analyses of interaction were used to assess if effects from gender or learning/attention problems on the CHQ scores differed between preterm and control children [26].

In the preterm group, simple linear regression analysis was used to study associations between CHQ scores and the following perinatal and neonatal variables: maternal infection, antenatal corticosteroids, birth weight ratio (ratio between birth weight and the 50^th. ^percentile for gestational age) and selected markers of early neonatal morbidity i.e. cerebral hemorrhage, days on mechanical ventilation, duration of supplemental oxygen and corticosteroid treatment for BPD.

A priori power calculation for this particular part of the overall follow-up study was difficult to perform, as the distribution of the variables of interest was not readily available for preterm children. By performing this study, we learned that one standard deviation (SD) for the psychosocial summary score was 10.3 for preterms and 4.8 for control subjects. With this information at hand, we have in retrospect calculated that the study had 80% power to detect group differences between preterm and control subjects of approximately 5.5 points, providing that the level of significance was set at 0.05. All statistical analyses were done with SPSS version 16/17 for Windows, except McNemar's test, which was done in StatXact.

## Results

### Subjects

All the 35 surviving and eligible preterms consented to participate in the study. On average, 1.3 potential control children had to be invited to find one willing match for each preterm index subject. All but two subjects (both born preterm) were Caucasians. Questionnaires were completed by 30 biological mothers, one foster mother and four fathers in the preterm group and by 31 biological mothers and four fathers in the control group.

### Demographic and clinical variables

Compared to the mothers of controls more preterm mothers had never married and were living single (4 vs. 1), while fewer parents of preterms had divorced or separated (1 vs. 4).

Within the preterm group, none had cerebral palsy or were blind or deaf, but 20%, (four boys and three girls) had minor impairments (Attention Deficit Hyperactivity Disorder, epilepsy, mild mental retardation or hearing impairment requiring hearing aid) (Table 1). All seven were living normal childhood lives, also reflected by their ability to take fairly complex instructions in relation to lung function testing and to complete a maximum exercise treadmill test.

**Table 1:** Neonatal and current clinical characteristics of the 35 children born extremely preterm

Number of boys, n (%)	13 (37)
Gestational age (weeks)^a)^	26.7 (1.7)
Birth weight (grams)^a)^	933 (204)
Impaired hearing, n (%)	2 (5.7)
Epilepsy, n (%)	3 (8.6)
ADHD ^b)^, n (%)	2 (5.7)
Mild mental retardation, n (%)	5 (14.3)
Intraventricular hemorrhage grade 1-2, n (%)	8 (22.9)
Maternal infection, n (%)	11 (31.4)
Prenatal steroid treatment, n (%)	15 (42.9)
Neonatal steroid treatment, n (%)	10 (28.6)
Days on ventilator ^a)^	8.3 (11.8)
Oxygen treatment (days)^a)^	57.4 (48.0)
Boys *	81.7 (59.9)
Girls *	43.0 (33.1)
Bronchopulmonal dysplasia	
-none; n (%)	9 (25.7)
-mild ^c)^; n (%)	14 (40.0)
-moderate/severe ^d)^; n (%)	12 (34.3)
Age when assessed (years) ^a)^	10.5 (0.4)

a) Mean (standard deviation)

b) Attention Deficit Hyperactivity Disorder

c) Requirement for oxygen treatment at age 28 postnatal days

d) Requirement for oxygen treatment at 36 weeks postmenstrual age

* Boys vs. girls p-value = 0.047 (independent sample t-test)

Compared to the term born controls, significantly more preterms had problems related to academic and social functioning (see additional file 1: results from paired tests on demographic variables). As many as 71% of the preterms vs. 20% of those born at term (odds ratio (OR): 7.0; 95% confidence intervals (CI): 2.2 to 27.6) had learning and/or attention problems, 38% vs. 3% (OR: infinite) were assessed to perform academically below average of their classmates and 65% vs. 20% (OR: 8.5; CI: 2.2 to 51.5) received academic and/or psychological support in school. The parents of 36% of the preterms vs. 3% of those born at term (OR: infinite) felt that they had received insufficient professional support when raising their children. Fewer preterm children participated in organized extracurricular physical activities, while participation in other social activities, such as choirs, bands, scouts, and various social clubs, did not differ between the groups see additional file 1.

### Child Health Questionnaire-PF50

**Figure 1:**
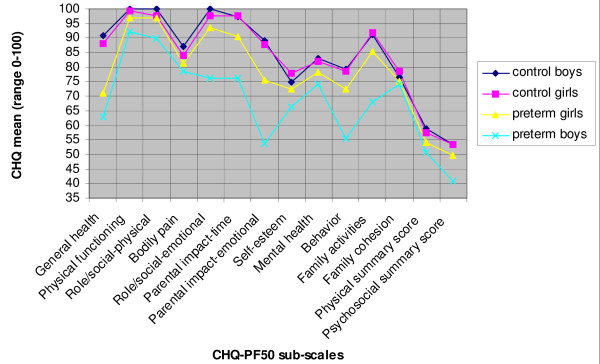
**Mean CHQ-PF 50^a) ^scores in matched pair data for 10 year old children born extremely preterm and controls born at term, according to gender**. a) Child Health Questionnaire-Parent Form 50; scale range 0-100 (except summary scores = norm based values with mean of 50 and standard deviation of 10).

Cronbach's alpha (internal consistency) for the sub-scales ranged from 0.70 to 0.94 for the two groups as a whole, except for mental health, which had an alpha of 0.55. In eight of the sub-scales and the two summary scores, parents of preterms scored their children significantly lower than did parents of control children (Figure 1). Preterm children were described as more juvenile and oppositional in their behavior (mean difference: -13; CI: -21.1 to -4.9), to have more limitations in role/social functioning due to behavior and emotional problems (mean difference: -11.4; CI: -20.0 to -3.0), and to have poorer general health (mean difference: -21.2; CI: -30.2 to -12.1). Their health and behavioral difficulties limited and interrupted family activities and caused more family tension (mean difference: -12.8, CI: -22.2 to -3.4). Compared to parents of term-born children, parents of preterms more often experienced emotional worries (mean difference: -21.0; CI: -31.1 to -11.0) and limitations in time available for personal needs (mean difference: -12.4; CI: -19.5 to -5.3) due to their children's physical and psychosocial health. However, relationships in general within the families (family cohesion) were assessed fairly equal in the two groups, as were the domains role/social physical functioning, bodily pain and self-esteem.

Differences between the preterm and term born children were mainly explained by the results for the preterm boys, and this modifying effect from gender was statistically significant (test of interaction) for four of the CHQ sub-scales as well as for the overall psychosocial summary scores (Table 2 and Figure 1). Learning and/or attention problems were associated with poorer HRQoL in all participants. However, this association was stronger for the preterms, particularly in areas related to general health perception and impact on parental time (Table 2), but there was no gender difference (tests of interaction, data not shown).

**Table 2:** Adjusted ^a) ^mean differences in CHQ-PF 50 ^b) ^scores between preterms and matched control children

CHQ sub-scales	Boys Mean (95% CI)	Girls Mean (95% CI)	Interaction p-value gender × preterm/control
Roles/social emotional	-21.7 (-35.1, -8.3)	-1.8 (-12.1, 8.5)	0.01
Parental impact-time	-19.4 (-31.3, -7.5)	-4.5 (-13.6, 4.6)	0.04
Parental impact-emotional	-25.8 (-41.5, -10.2)	-4.1 (-16.1, 7.9)	0.02
Behavior	-22.3 (-35.6, -9.1)	-4.5 (-14.7, 5.7)	0.02
Psychosocial summary score	-11.0 (-17.9, -4.1)	-2.2 (-7.5, 3.0)	0.03

	**With learning or attention problems** **Mean (95% CI)**	**Without learning or attention problems** **Mean (95% CI)**	**Interaction p-value attention/learning × preterm/control**

General health	-20.5 (-31.7, -9.3)	-2.0 (-16.1, 12.2)	0.02
Parental impact-time	-17.1 (-26.5, -7.8)	2.9 (-9.6, 15.4)	0.01

	**Adjusted ^a) ^results without interaction** **Mean (95% CI)**		

Physical functioning	-3.9 (-8.1, 0.4)		
Role/social physical	1.4 (-6.2, 9.0)		
Bodily pain	5.0 (-5.2, 15.3)		
Self-esteem	-3.4 (-12.3, 5.5)		
Mental health	-4.0 (-9.6, 1.8)		
Family activities	-9.2 (-19.3, 0.9)		
Family cohesion^c)^	-8.7 (-19.7, 2.3)		
Physical summary score	-1.4 (-5.3, 2.4)		

a) Results are presented adjusted for gender, physical activity, learning and/or attention problems and forced expiratory volume in the first second (% predicted), including the interaction term when this was significant.

b) Child Health Questionnaire-Parent Form 50; scale range 0-100 (except summary scores = norm based values with mean of 50 and standard deviation of 10)

c) Single item score

There were differences between the preterm and the term born control group with respect to participation in organized physical activity, maternal education and lung function (FEV_1_) see additional file 1. These potential confounding factors did not alter the conclusions from the regression models.

Within the preterm group, those who had received neonatal steroid treatment for BPD scored poorer in the domain for role/social functioning related to emotional and behavioral problems (p = 0.027). Otherwise, none of the assessed perinatal or neonatal variables significantly influenced subsequent quality of life scores (simple linear regression). The boys required more days of oxygen treatment than girls (Table 1). In a multiple linear regression model, male gender and not neonatal oxygen treatment, was significantly associated with poor HRQoL outcomes (data not shown).

## Discussion

Being born extremely preterm was associated with inferior health related quality of life at the age of ten, particularly for the boys. Nearly three out of four preterms had problems related to school performance, compared to one out of five born at term. Academic concerns were related to quality of life in all participants, but more strongly in preterms.

### Strengths and limitations

The major strengths of this study were the population-based design and the complete participation. Since there were no subjects with major impairments, there were no exclusions in the analyses, increasing the study's validity for prematurely born children expected to follow a normal social progress during childhood. On average, only 1.3 term born subjects had to be approached to recruit a complete control population, limiting potential sample bias. The same team conducted all parts of the study, limiting inter-observer variability. The major weakness of the study was the relatively low overall number of participants, which made it susceptible to statistical type II errors and thus weakening particularly negative conclusions. However, the reported associations were marked and consistent, and appeared statistically robust. Control subjects were selected with the intention to create a group as similar to the preterm group as possible, with one exception only, the gestational age at birth. Preterm birth has been associated with socioeconomic shortcomings [27], and one may argue that a control population should reflect this. However, the Norwegian society is characterized by a fairly egalitarian sociodemographic structure, and therefore we opted to match control subjects on gender and the timing of birth only. In this study, we observed a tendency for a lower educational level in mothers of preterms compared to mothers of control subjects, but no such tendency for the fathers. These factors did not influence the conclusions of the study.

Knowledge about HRQoL in school-aged children who were born extremely preterm is relatively scarce. Assessment of a subjective phenomenon like quality of life through information provided by others, in this study the parents, has limitations. However, when self-reported data are difficult or impossible to obtain, this is a valid method to generate information [9,13]. Also, parental reports will reflect the challenges of these children and of their families such as they are perceived by the most important person in the life of a child - the parent.

The potential burden of raising a preterm child starts the very minute the parent(s) leave the NICU. Thereafter, a continuously changing panorama of new circumstances and potential difficulties will materialize with the growth of the child, challenging the family structure and its members. A positive finding from the present and similar studies was that parents of the preterm children reported overall family relationships to be good[10,28], and that fewer parents in the preterm group had divorced. These findings seemingly contradict the observed CHQ-PF50 scores, which indicate an increased burden of parenting. This result suggests that some forms of adjustment, acceptance, or coping mechanisms are activated within the family by the uncertainty of raising these children. One third of preterm parents reported insufficient societal professional support. Recent reports from the USA and Denmark support this finding [29,30]. Lack of professional support may be another factor increasing the observed burden of parenting. Alternatively, there may be inherent challenges involved in the process of parenting many preterm children, making it difficult to offer or receive outside help. It is of considerable interest in this context that two quite different social welfare systems, namely those of Norway and the USA, both seem to fail in providing adequate help for these families.

The school is an arena of utmost importance for both academic and social success in life. As preterm children were reported to have more learning difficulties and/or attention problems, they naturally received more support, both academically and psychologically. In a previous study from our institution, eleven year old children with birth weights less than 2000 g without major disabilities had twice as many school problems and were referred to the School Psychological Services two to three times more often than children born at term [31]. In the present study, this ratio approached four times that of their matched peers born at term, probably because our preterm cohort was more immature at birth. Similar concerns with respect to academic performance have also been expressed by others examining populations relatively similar to ours [32-34]. The observed association between learning and/or attention problems at school and quality of life was present in all participating subjects, but was more prominent among those born preterm. Why academic shortcomings had a greater negative influence on the quality of life in children born preterm and their parents cannot be answered within the frame of this study since we did not assess the nature and the extent of the learning and attention problems.

Physical activities and sports are important elements of a normal childhood, influencing subsequent physical as well as social development. Neurosensory and cognitive abilities, neuromotor skills, aerobic capacity and personal ambitions influence the extent of individual success. Compared to term born controls, the preterms took less part in physical activities and sports, while they participated to a similar extent in other nonphysical, extracurricular activities. We are not aware that others have reported this pattern. Typical features of children born preterm, e.g. a sense of insecurity, clumsiness, attention problems and reduced physical capacity [3,35,36] may limit their ability and subsequent interest in physical activities. Participation in non-physical social activities might provide an important alternative arena for psychosocial training. Considering the well described tendency towards behavioral problems and reduced social competence in this group of children [4,37-39], this finding is encouraging. At the age of ten, parents have a strong influence on the choice of activities and lifestyle of their children and one explanation may be that parents of preterms acknowledge their children's physical limitations and therefore encourage them to take part in activities felt to be appropriate and within their physical and mental abilities. A contributing factor to the high rate of participation in non-physical social activities might be that at this age the full impact from potential limitations was not sufficiently obvious to discourage participation.

The excess of concerns and poorer HRQoL scores among the preterm children were mainly explained by the poorer results for the boys. This finding is in line with previous studies on preterm subjects, but contradicts similar studies in unselected populations of similar ages [10,40,41]. Male gender is a well known risk factor for neonatal mortality and morbidity in preterm neonates. In the present study, the boys had a neonatal history characterized by nearly twice as many days of oxygen supplementation compared to the girls. Statistical handling of this situation is difficult, i.e. which is the "true" explanatory factor: gender or prolonged oxygen requirements. However, within the frame of the present study, male gender and not neonatal oxygen treatment appeared as the most important and most robust explanatory variable. It has been suggested that a poorer prognosis in terms of survival and early morbidity for boys also extends to their later development, even for survivors without major disabilities [42,43]. Hintz et al. propose that there may be a gap in the societal support offered to boys in their first two years of life [30]. Why males are more vulnerable than females may partly be explained by a biological fragility of the male fetus, possibly reinforced by an attitude from society that boys are, or must be mademore resilient than girls, thus adding "a social insult to the biologicalinjury" (S. Kraemer p. 1609) [44]. Based on the present study, one might suggest that this should have implications also for the clinical management of males in a NICU setting, as well as for the upbringing of male children born extremely preterm.

Within the preterm group, subjects who had received neonatal corticosteroid treatment scored poorer in the domain of social functioning. In this context one must bear in mind that corticosteroids are used to treat severely ill neonates with a number of potential risk factors for poor outcome. However, the observation agrees with an accumulating number of reports that neonatal treatment with corticosteroids is associated with an increased risk of impairments [45]. Apart from this notable exception, there were no associations between the assessed neonatal and perinatal variables and subsequent HRQoL outcomes. This contradicts findings reported by our own group and by others regarding physical outcomes such as lung function and pulmonary CT scans [6,46]. One may argue that impact from a diverse postnatal environment will influence multidimensional outcomes such as HRQoL more than unidimensional physical outcomes. Additionally, the neonatal history of extreme preterms varies considerably and most medical problems somehow tend to be interrelated, complicating research on subsequent cause and effect relationships. Also, the limited sample size may have precluded our ability to detect potential associations that might be present. In fact, neonatal treatment with corticosteroids has been reported to have an adverse effect on academic achievement at the age of eight and maternal infection has been reported to predict neurodevelopmental impairments [2,47]. Randomized long-term follow-up studies must be performed to explore these issues.

## Conclusion

Being born extremely preterm was associated with inferior HRQoL at the age of ten, particularly for boys, affecting the child as well as the family. The majority of parents of preterms reported that their children had learning and/or attention problems, and one third experienced insufficient professional support. Learning and/or attention problems at school were associated with inferior HROoL in all participants, but this association was stronger among preterms. Treatment and support offered to preterm children and their families needs to be addressed in future studies, particularly if the child is a boy.

## Competing interests

The authors declare that they have no competing interests.

## Authors' contributions

BV contributed to the study design, statistical analysis, and interpretation of the data and drafting of the manuscript. TM contributed to interpretation of the data, drafting and critical revision of the manuscript. GEE contributed with statistical expertise and analysis, and interpretation of the data and critical revision of the manuscript. MG contributed to interpretation of the data, drafting and critical revision of the manuscript. TH contributed to the study design, the data collection, interpretation of the data, and drafting and critical revision of the manuscript. All the authors have given final approval of the submitted manuscript.

## Supplementary Material

Additional file 1 **Current clinical and sociodemographic characteristics of the preterm cohort and their matched controls**. The data provided represent the statistical analysis of McNemar, a non-parametric method, and t-test for paired samples to explore group differences between preterm and matched control children on clinical and sociodemographic characteristics. Click here for file
