# Supplementary material for: Health related quality of life after extremely preterm birth: a matched controlled cohort study
Source: Health Qual Life Outcomes. 2010 May 23;8:53. doi: 10.1186/1477-7525-8-53 (PMC2894784; doi:10.1186/1477-7525-8-53)
Supplement: Additional file 1 — Current clinical and sociodemographic characteristics of the preterm cohort and their matched controls. The data provided represent the statistical analysis of McNemar, a non-parametric method, and t-test for paired samples to explore group differences between preterm and matched control children on clinical and sociodemographic characteristics. [file 1477-7525-8-53-S1.PDF]

Additional file. Current clinical and sociodemographic characteristics of the preterm cohort and their matched controls

	Premature n (%)	Control n (%)	n_1/n_2 [a)]	Odds ratio (95% confidence interval)	p-value [b)]	Missing: preterms /controls
Reading and writing difficulties	12 (36.4)	5 (14.3)	10/2	5.0 (1.2, 31.8)	0.03	2/0
- Girls	6 (28.6)	4 (18.2)				
- Boys	6 (50.0)	1 (7.7)				
Learning and/or attention problems	25 (71.4)	7 (20.0)	21/3	7.0 (2.2, 27.6)	<0.001	0/0
- Girls	14 (63.6)	3 (13.6)				
- Boys	11 (84.6)	4 (30.8)				
Academic and/or psychological support	22 (64.7)	7 (20.0)	17/2	8.5 (2.2, 51.5)	<0.001	1/0
- Girls	13 (59.1)	5 (22.7)				
- Boys	9 (75.0)	2 (15.4)				
School achievement below average of classmates	13 (38.2)	1 (2.9)	12/0	Inf [c)] (3.2, Inf)	<0.001	1/1
- Girls	5 (22.7)	0 (0.0)				
- Boys	8 (66.7)	1 (7.7)				
Basic or attendance benefit [d]	5 (15.6)	0 (0.0)	5/0	Inf (1.0, Inf)	0.06	3/1
- Girls	1 (4.8)	0 (0.0)				
- Boys	4 (36.4)	0 (0.0)				

Inadequate professional support during childhood	11 (35.5)	1 (3.0)	10/0	Inf (2.7, Inf)	0.002	4/2
- Girls	7 (35.0)	1 (5.0)				
- Boys	4 (36.4)	0 (0.0)				
No organized physical activity	17 (48.6)	7(21.2)	13/4	3.2 (1.0, 10.8)	0.04	0/2
- Girls	11 (50.0)	4 (19.0)				
- Boys	6 (46.2)	3 (25.0)				
No organized social activity (physical excluded)	21 (63.6)	22 (64.7)	5/5	1.0 (0.3, 3.5)	1.00	2/1
- Girls	12 (57.1)	12 (57.1)				
- Boys	9 (75.0)	10 (76.9)				
Mothers' education: below college/university	29 (85.3)	19 (54.3)	12/2	6.0 (1.4, 37.5)	0.01	1/0
- Girls	18 (81.8)	13 (59.1)				
- Boys	11 (91.7)	6 (46.2)				
Fathers' education: below college/university	24 (72.7)	21 (60.0)	9/5	1.8 (0.6, 5.5)	0.42	2/0
- Girls	16 (72.7)	12 (54.5)				
- Boys	8 (66.7)	9 (69.2)				
$FEV_1\%$[e)], mean (SD)	79.2 (9.1)	87.0 (8.7)			0.001 [f)]	0/0
- Girls	79.8 (10.3)	85.7 (9.5)				
- Boys	78.2 (6.8)	89.3 (7.0)				

a) Ratio of discordant pairs, n_1 = preterm, n_2 = control

b) McNemar's test, preterms vs. controls

c) Inf = Infinite

d) From the national compulsory health insurance scheme

e) FEV_1: Forced expiratory volume in the first second (% predicted)

f) Paired sample t-test
